# Clinical and radiographic evaluation of biodentine versus calcium hydroxide in primary teeth pulpotomies: a retrospective study

**DOI:** 10.1186/s12903-018-0522-6

**Published:** 2018-04-02

**Authors:** Silvia Caruso, Teresa Dinoi, Giuseppe Marzo, Vincenzo Campanella, Maria Rita Giuca, Roberto Gatto, Marco Pasini

**Affiliations:** 1 Department of Life, Health and Environmental Sciences, University of L’Aquila, L’Aquila, Italy; 2 Department of Clinical Sciences and Translational Medicine, University of Roma Tor Vergata, Rome, Italy; 3 Department of Surgical, Medical, Molecular Pathology and Critical Area, Unit of Pediatric Dentistry, University of Pisa, Pisa, Italy

**Keywords:** Pulpotomy, Primary teeth, Biodentine, Calcium hydroxide

## Abstract

**Background:**

Pulpotomy is the surgical removal of the entire coronal pulp with preservation of the radicular pulp vitality. The purpose of this retrospective study was to evaluate the clinical and radiographic success of pulpotomy of primary molars using two materials, biodentine and calcium hydroxide.

**Methods:**

Records of 400 primary molars in 360 paediatric participants (mean age: 7.5 ± 1.6 years, ranging from 5 to 9 years) with dental caries who required pulp therapy were included in this study. Biodentine was used on 200 teeth, and calcium hydroxide (CH) was used on another 200 teeth, as a pulpotomy material. Clinical and radiographic evaluation was performed after 9 and 18 months. Statistical analysis was evaluated with the chi-squared test, and the level of significance was set at *p* < 0.05.

**Results:**

The treatment success with CH was 85.5% after 9 months and 79.5% after 18 months, while the success rate of biodentine was 94% after 9 months and 89.5% after 18 months. The statistical analysis with the Chi-squared test showed that the clinical and radiographic success rate with biodentine was significantly higher than CH (*p* < 0.05).

**Conclusions:**

Biodentine exhibited a higher clinical and radiographic success rate compared to CH. However, besides the clinical results, biodentine has some disadvantages, such as higher costs, compared to CH.

## Background

The maintenance in the arch of a primary tooth with a deep caries extending up to the pulp is particularly important to maintain the space, drive the eruption of the permanent tooth and help children chew food properly and speak more clearly [1]. The execution of an endodontic therapy of a primary tooth helps prevent avulsion, requiring, in some cases, the application of a space maintainer to prevent unwanted movement of the neighbouring teeth and consequent loss of space in the arch [2]. Moreover, premature tooth loss can lead to malocclusion with aesthetic, phonetic and functional problems that may be transient or permanent [3].

Pulpotomy is a therapy that involves removal of the pulp from the pulp chamber of the primary tooth without removal of the canal pulp as well as the application of a medication at the entrance of the root canal to fix or stimulate the repair of the vital remaining pulp. Pulpotomy is indicated in cases of exposed vital pulps by the caries process, by accident during cavity preparation, or as a result of injury and fracture of the tooth in primary teeth [4]. However, it is not indicated for primary teeth with internal resorption, furcal perforation, insufficient root structure, and periradicular pathosis that may alter permanent successor eruption [5]. Furthermore, the partial removal of carious dentin is the currently indicated technique in extensive caries lesions. In shallow to moderate dentinal cavitated caries lesions (that radiographically appear to extend less than 75% into the dentin), this technique is often used without the risk of exposing the pulp [6].

Several materials, such as dressing formocresol, glutaraldehyde, calcium hydroxide, ferric sulfate, iodoform, and MTA (Mineral Trioxide Aggregate), have been suggested over the years.

The formocresol, introduced by Bucley in the form of 19% formaldehyde and 35% of tricresol in an aqueous solution of glycerin and water, was the first material to be used and allowed to mummify the entire residual pulp, but it remains a potentially toxic material [7]. Other materials, such as calcium hydroxide, facilitate the healing of the pulp by creating a hard tissue at the orifice, while the use of ferric sulfate helps generate a complex of ferric ions in contact with the blood and promotes hemostasis [8]. MTA, introduced in 1995, is a material that creates a bridge of dentine in a biocompatible way; it has excellent sealing ability, and there are bone morphogenic proteins and growth factors that act through their osteogenic potential in pulp repair [9].

In addition, electrocautery and removal of the pulp by laser techniques have the advantage of obtaining good control of bleeding, although there is weak evidence of tissue repair.

Several authors studied the success rate of the different materials, in terms of clinical and radiological findings, for pulpotomy in primary teeth; MTA is currently considered an optimum material for vital pulp therapy and in the medium-term clinical assessment because it has a high success rate. Yildiz and Tosun [10] evaluated four pulpotomy treatments in primary molars and found that after 30 months, the clinical success rates were 100% for MTA, 95.2% for formocresol, 96.4% for ferric sulfate, and 85% for calcium hydroxide.

Grewal et al. [11] compared biodentine and calcium hydroxide and found that primary teeth treated with biodentine showed a favourable regenerative potential along with clinical success compared to the children treated with calcium hydroxide.

Sirohi et al. [12] compared the clinical and radiographic success rates of biodentine and ferric sulfate and found after 9 months, 96% clinical success rate in the ferric sulfate and 100% in the biodentine group; furthermore, radiographic success rate in the ferric sulfate group was slightly lower (84%) than the success rate found in the biodentine group (92%).

Biodentine is a calcium silicate cement that can be used for pulpotomy because it is very successful in the formation of a dentine bridge. Additionally, it is mechanically stronger and less soluble and produces tighter seals than calcium hydroxide; moreover, it avoids the drawbacks of MTA, i.e., extended setting time, difficult handling characteristics and high cost [13].

The purpose of this retrospective study is to evaluate the success in the medium- to long-term clinical and radiological pulpotomy of the second primary molars by comparing two different materials, biodentine and calcium hydroxide, that are traditionally used for pulpotomy.

## Methods

### Patient records

A search of patient records was conducted to identify all primary tooth pulpotomy treatments. Inclusion criteria for this study were the following: children in good general health with no systemic diseases and no history of taking medicines for chronic therapies, second primary molars with exposure of the pulp as a result of dental caries, no degeneration of pulp, and no excessive bleeding (bleeding from the root canals had to stop within 5 min with cotton soaked in sterile saline pellets) as well as no clinical symptoms, such as pathologic mobility, swelling, or pain on percussion. In addition, the teeth had to lack internal or external root resorption or destruction of the periradicular bone tissue according to radiography (endoral radiographs). Finally, the teeth had to be recoverable by a composite reconstruction. The molars with fractures or infiltration of the composite restoration, performed at the end of pulpotomy, which may lead to failure of endodontic therapy, were excluded from this study. The study was conducted at the paediatric dentistry unit of the University of L’Aquila (Italy) between January and October 2016.

The Ethics Committee approved this study.

The participants were selected from the records of the subjects referred to the university paediatric dentistry clinic.

### Clinical procedures and clinical and radiographic evaluation

A single dentist performed the pulpotomy under the same clinical conditions and by using the same tools. The procedure consisted of anaesthesia of the tooth with mepivacaine without adrenaline; application of a rubber dam; removal of dental caries and pulp with a round diamond bur (#6), which was used at high speed and with adequate water cooling; debris removal with sterile water; control of bleeding with cotton pellets soaked in saline and applying slight pressure with additional dry pellets until the cessation of bleeding. Excessive air on the exposed pulp, which may cause tissue desiccation, was avoided.

At this point, the medication was applied to the pulp at the level of the root canals.

Patient records of 200 primary molars treated with calcium hydroxide and 200 primary molars treated with biodentine were analysed. Application of the medication was randomly chosen for the teeth.

Application of calcium hydroxide (Calxyl®, OCO preparate, Dirmstein, Germany) was performed with a sterile calcium hydroxide powder freshly mixed with distilled water, which was applied to the radicular pulp and gently adapted with a cotton pledget [14].

Biodentine (BIODENTINE®, Septodont, Saint-Maur-des-Fossés, France) contains tricalcium silicate, dicalcium silicate, calcium carbonate and oxide filler, shade iron oxide, and zirconium oxide; tricalcium silicate and dicalcium silicate are, respectively, indicated as the main and second core materials, while zirconium oxide serves as a radiopacifier. The material was applied on the inlet of the root canals using a steel spatula and distributed with a condenser amalgam. After 12 min, the material self-hardened.

The cavities were restored with a chemically cured glass ionomer cement (Vidrion, SS White), adhesive (Scothbond Multipurpose, 3 M ESPE) and direct restoration of light-cured composite material (Venus Pearl, Heraeus Kulzer).

A single operator performed all pulpotomies, while a blind investigator, who was unaware of group assignment and had received extensive training, performed all clinical and radiographic examinations. The records of each patient included the following: number of teeth, pulpotomy procedures, clinical and radiographic pre- treatment and post-treatment conditions of teeth, and the date of treatment and follow-ups.

The follow-ups were performed after 9 months and 18 months for clinical and radiographic evaluation; radiographic evaluation was performed by digital intraoral X-rays (Kodak 2100) with the parallel ray technique, Rinn centring and a 2× magnification viewer.

The clinical parameters for evaluating the therapeutic success were the absence of spontaneous pain, tenderness at percussion, swelling and pathologic mobility. In addition, the radiological parameters of success were the absence of exfoliation, flaring of the periodontal ligament space, internal or external root resorption and radicular radiolucency.

The obliteration of the pulp at the level of the root canals was not considered among the success parameters.

### Sample size

Calculation of the sample size was based on previous studies that found clinical and radiographic success of 94.73% for teeth treated with biodentine [13], while the clinical and radiographic success rates calcium hydroxide ranged between 80 and 90% [10].

The predetermined sample size calculation to obtain a 90% power at a 5% level of statistical significance and beta of 0.1 was 200 teeth for each group.

### Measurements reproducibility

The calibration process included intra and inter-examiner reproducibility.

Intra-observer analysis was calculated evaluating all radiograms twice after 1 week.

The degree of agreement was quantified by kappa (k) and a k score of 0.85 was obtained.

As regards the inter-examiner reliability, the calibration process of radiographic evaluations was performed by two paediatric dentists, who had experience in endodontic treatments, which were standardized prior to the analysis; Cohen’s kappa (κ) was calculated, and a value of 0.87 was obtained.

### Statistical analysis

The statistical analysis with the Chi-squared test and odds ratio, using logistic regression with one predictor variable (age) for each dependent variable, was conducted to evaluate intragroup significant differences between the two time points and inter-group differences at 9 and 18 months. All data were analysed with Stata software (version12; StataCorp, College Station, Tex), and the level of significance was set at *p* < 0.05.

## Results

The study was conducted on a total of 400 primary molars in 360 children (189 males and 171 females with a mean age of 7.5 ± 1.6 years and range from 5 to 9 years). The calcium hydroxide (CH) group included 94 males and 85 females (mean age: 7.6 ± 1.4 years) and the biodentine group included 95 males and 86 females (mean age: 7.4 ± 1.6 years).

All clinical and radiographic results are shown in Table 1.

**Table 1:** Intra-group and inter-group differences in the first follow up (from the beginning of the treatment to 9 months) and in the second follow-up (from the beginning of the treatment to 18 months)

	Calcium hydroxide 9 months	Biodentine 9 months	Calcium hydroxide 18 months	Biodentine 18 months	Intra-group *p*- value CH (OR: CI 95%)	Intra-group *p*- value Biodentine OR CI 95%	Inter-group *p*- value 9 months OR CI 95%	Inter-group *p*- value 18 months OR CI 95%
Sex	91 males, 80 females,	98 males, 90 females,	88 males, 75 females,	94 males, 85 females,				
Age (years)	7.7 ± 1.6	7.5 ± 1.5	7.5 ± 1.5	7.6 ± 1.7				
Total success	171 (85.5%)	188 (94%)	163 (81.5%)	179 (89.5%)	0.28 (1.34: 0.79–2.28)	0.1 (1.84: 0.88–3.85)	0.005* (0.38: 0.19–0.76)	0.02* (0.52: 0.29–0.92)
Clinical success	182 (91%)	195 (97.5%)	179 (89.5%)	193 (96.5%)	0.61 (1.19: 0.61–2.3)	0.56 (1.41:0.44–4.53)	0.005* (0.26: 0.09–0.71)	0.006* (0.31: 0.13–0.74)
Radiographic success	171 (85.5%)	188 (94%)	166 (83%)	181 (90.5%)	0.49 (1.21:0.7–2.07)	0.19 (1.64:0.78–3.48)	0.005* (0.38: 0.19–0.76)	0.027* (0.51: 0.28–0.93)
Pain	11 (5.5%)	2 (1%)	13 (6.5%)	3 (1.5%)	0.67 (0.84:0.37–1.92)	0.65 (0.66:0.11–4.01)	0.011* (5.76:1.26–26.34)	0.01* (4.57: 1.28–16.28)
Percussion	9 (4.5%)	1 (0.5%)	9 (4.5%)	2 (1%)	1 (1:0.39–2.57)	0.56 (0.5:0.04–5.53)	0.01* (9.38:1.18–74.72)	0.03* (4.66: 1–21.87)
Swelling	12 (6%)	1 (0.5%)	12 (6%)	1 (0.5%)	1 (1: 0.44–2.28)	1 (1:0.06–16.1)	0.002* (12.7:1.64–98.64)	0.002* (12.7: 1.64–98.64)
Mobility	2 (1%)	1 (0.5%)	3 (1.5%)	1 (0.5%)	0.65 (0.11–4.01)	1 (1:0.06–16.1)	0.56 (2.01:0.18–22.35)	0.32 (3.03:0.31–29.38)
Esfoliation	16 (8%)	6 (3%)	18 (9%)	9 (4.5%)	0.72 (0.88:0.43–1.78)	0.43 (0.66:0.23–1.88)	0.028* (2.81:1.08–7.34)	0.07 (2.1:0.92–4.79)
Widening	13 (6.5%)	5 (2.5%)	16 (8%)	8 (4%)	0.56 (0.8:0.37–1.71)	0.4 (0.62:0.2–1.91)	0.054* (2.71: 0.95–7.75)	0.09 (2.09:0.87–4.99)
Resorption	14 (7%)	5 (2.5%)	17 (8.5%)	8 (4%)	0.57 (0.81:0.39–1.69)	0.4 (0.62:0.2–1.91)	0.034* (2.94:1.04–8.31)	0.06 (2.23:0.94–5.29)
Radiolucency	2 (1%)	2 (1%)	0 (0%)	3 (1.5%)	0.16 (0)	0.65 (0.66:0.11–4.01)	1 (1:0.14–7.17)	0.08 (0)

Chi-square test *p < 0.05

In the CH-group, after 9 months, a clinical and radiographic failure rate was found. It was observed that all teeth with a clinical failure also had at least one radiographic sign of failure. The total success rate after 18 months was slightly lower compared to the first follow-up. The intra-group difference between the two follow-ups (0–9 and 0–18 months) showed that the total clinical and total radiographic failure rates were not significantly different (*p* > 0.05).

Regarding the group treated with biodentine, at 9 months, a low clinical and radiographic failure rate was observed. All teeth with clinical failure also had at least one radiographic sign of failure. The follow-up after 18 months showed slight clinical and radiographic failure percentages, and the intragroup difference between the two follow-ups was not significantly different (*p* > 0.05).

Moreover, in both groups, no significant intra-group difference (*p* > 0.05) was detected for each clinical and radiographic parameter between the two follow-ups.

The statistical analysis showed that the success percentage in the biodentine group was significantly higher compared to the CH group at 9 months (*p* < 0.05). Similarly, in terms of the total success rate from the beginning of the treatment to 18 months, there was a significant difference in the total success rate between the two groups (*p* < 0.05).

Clinical failure was significantly higher in the CH group compared to the Biodentine group at 9 months (*p* < 0.05) and from the beginning of the treatment to 18 months (*p* < 0.05).

In addition, radiographic failure was significantly higher in the CH group compared to Biodentine at 9 months (*p* < 0.05) and from the beginning of treatment to 18 months (*p* < 0.05).

At 9 months, significant (*p* < 0.05) inter-group differences were found for all clinical and radiographic parameters, except for the mobility and radiolucency, which were similar in the two groups.

After 18 months, only pain, percussion and swelling were significantly (*p* < 0.05) higher in the CH group compared to the biodentine group.

## Discussion

The main findings of the present study showed that clinical and radiographic failures were lower in the group treated with biodentine in comparison to the group treated with CH. Furthermore, it was observed that most of the failures occurred in the first follow-up period and, for this reason, clinical and radiographic controls should be performed at regular intervals after pulpotomy treatment in primary molars.

Pulpotomy procedures consist of the use of a material that allows the isolation of the root canal pulp before proceeding with the final restoration; this material must be biocompatible, be antibacterial and have stable dimensions [15].

In the literature, several biomaterials for pulpotomy have been discussed and the therapeutic clinical procedure is likely more important than the type of material [16]. In addition, the diagnostic phase is a key element because the teeth that need pulpectomy cannot be treated with pulpotomy techniques [17].

Biodentine has characteristics similar to natural dentin and enables the stimulation of growth factors that activate dentinogenesis and differentiation of odontoblasts. It has been stated that biodentine has bioactive properties, encourages hard tissue regeneration, and provokes no signs of moderate or severe pulp inflammation response [18]. CH, due to its high pH, neutralizes acids and stimulates odontoblasts, favouring healing and inducing the formation of hard tissue (dentinated bridges and apical closures) [19, 20].

Another important factor affecting the therapeutic success is the absence of microleakage and sealing ability. Nowicka et al. [21] showed that the biodentine can prevent microleakage and the sealing ability of CH is well known because it was one of the first materials used for pulpotomy.

In the present study, restoration with composite material was preferred to the use of amalgam and steel crowns because it was possible to better isolate the teeth from saliva and to perform adhesive reconstruction that provides a good aesthetic result with suitable closure. In fact, Guelmann et al. showed that there were no significant differences in terms of the success in teeth treated with steel crowns and teeth treated with conservative restorations [22].

Data from the present study showed that the two materials were significantly different in terms of the clinical and radiographic outcomes after medium- and long-term evaluation; biodentine exhibited a higher success rate. Regarding the clinical relevance, biodentine exhibits good material handling and performance with a high compressive strength to external forces; however, the main disadvantages of biodentine include the setting time and material cost. Compared to CH, biodentine does not have high cost effectiveness; furthermore, the setting time of biodentine is higher than CH at between 9 and 12 min.

In our study, the majority of failures occurred at the first follow-up, while the second control had a lower percentage of later failures in both groups. This suggests that if the therapeutic procedure shows no signs of failure in the first nine months, the percentage of those teeth that will show signs of failure is decreased.

Regardless of the type of material, we detected a low rate of failure in both the clinical and radiographic controls in both groups. Kusum et al. obtained a radiographic success rate of 80% and clinical success rate of 100% after 9 months with biodentine. The same authors observed a higher percentage in both the radiographic control when using MTA (92%) and clinical control (100%), while lower percentages were observed when using propolis, which showed a 72% radiographic success and 80% clinical success after 9 months [23]. In another study, the success of pulpotomy with biodentine and restoration with steel crowns was higher with 97% clinical success and 95% radiographic success in the follow-up after 12-months [24]. El Meligy et al. compared the success rates of biodentine and formocresol and found a 100% success rate for both treatments; however, the follow-ups in their study were shorter (3 and 6 months) [25].

After 18 months, the clinical and radiographic success rates of CH were, respectively, 82.3 and 76.5%, which are similar to those found in our study [14].

In the review by Yousef H Al-Dlaigan, who analysed the success rate of the various pulp treatments, there is a success rate of 80% for CH with varying percentages depending on the follow-up and number of involved teeth [26].

In a previous study it was observed that internal resorption was the most common finding, due to the morphology and thinness of molar roots [27], while, in our study, esfoliation, root resorption and widening of the periodontal ligament space showed similar percentages.

As discussed by Markovic et al., histological evaluation of the teeth is only relevant for diagnosis, but it is mostly based on evaluation of extracted teeth that failed pulpotomy treatment, making it difficult to evaluate the pulp response to treatments [14].

The relevance of the present study is that the total success of pulpotomy with biodentine is 8.5 and 8% more than CH after 9 and 18 months, respectively. Regarding the clinical success, the percentages are slightly lower, at 6.5 and 6% after 9 and 18 months, respectively. The different success rates between the two materials are due to a higher clinical and radiographic failure rate, which was only recorded in the CH group during the first 9 months of follow-up after therapy. Between the 9th and 18th month, a similar failure rate was observed in the two groups.

The failure of therapeutic treatment in primary molars in the present study may be from a wrong diagnosis (pulpotomy instead of pulpectomy). The failure may also be due to imperfect control of haemostasis during treatment or the appearance of microleakage in restorations that was not detected by the operators.

Radiographic failures were higher than clinical ones and for this reason it is important to perform a radiographic evaluation of treated teeth even in absence of clinical signs. The recommendation of the study is that the choice of the correct medication may slightly decrease the failure rate of pulpotomies in primary teeth. However, correct diagnosis and correct clinical procedures are necessary in addition to the treatment type. The cost-effectiveness of the material should also be considered by the operator for the treatment choice. Finally, the operator should limit pulpotomies to those that are strictly necessary because the partial removal of carious dentin is a more conservative technique that is indicated in extensive caries lesions [6].

It is important to study the use of biodentine in pulpotomy as different researches have shown the ability of this material to induce cell proliferation and biomineralization and its ability to induce pulp repair and dentin synthesis through an increase of transforming growth factor-31 [12]. Moreover, biodentine does not need the use of a separate restoration because has high strenght and an optimal marginal adaptation as observed by Koubi et al. [28].

The present study adds important insight into the process of choosing the right medication for primary molar pulpotomies. Only few previous studies, in scientific literature, analysed the clinical and radiographic failures of biodentine with short follow-ups and including small samples [12, 25], while in this study a longer follow-up a larger sample of participants were considered.

The limitations of the study included the absence of a very short-term follow-up; for this reason, we cannot exactly determine when failures occurred in the first 9 months. Additionally, an additional follow-up after 18 months could help with evaluating the success rates in the following period. We have limited our analysis to only two medications, but other materials, such as MTA, could be included in further studies to compare the success rates of different medications. Moreover, the modifications in the regenerative properties of the pulp can be controlled by narrowing the age range of the included participants.

For these reasons, further studies will be necessary to confirm the findings of the present study.

## Conclusions

The findings of this study show that biodentine exhibits a higher clinical and radiographic success after 9 and 18 months compared to CH. These results suggest the potential of biodentine for being used as a pulpotomy medicament in primary teeth.

Most clinical and radiographic failures occurred in the first 9 months after pulpotomy in both groups so we can state that the failures of pulpotomy treatment in primary teeth were more prone to occur in a short time; this suggests that if pulpotomy shows no signs of failure in the first 9 months, the percentage of teeth that will show signs of clinical or radiographic failure is decreased. For this reason, periodic follow-up should be performed more often during the first 9 months after the treatment.

However, beyond the clinical results, biodentine has disadvantages such as higher costs and a longer setting time compared to CH. Clinical and radiographic evaluations should be performed carefully by the pediatric dentist to achieve correct diagnosis and both materials (CH and biodentine) can be used successfully for pulpotomy in primary molars.

## Abbreviations

CH: Calcium hydroxide
CI: Confidence interval
OR: Odds ratio
